# Elevated risk of patellofemoral osteoarthritis following ACL reconstruction compared to contralateral knees: A systematic review and meta‐analysis

**DOI:** 10.1002/jeo2.70467

**Published:** 2025-11-18

**Authors:** Domenico Franco, Alexander Bumberger, Chilan B. G. Leite, Sebastian Schmidt, Rocco Papalia, Vincenzo Denaro, Cale Jacobs, Christian Lattermann

**Affiliations:** ^1^ Department of Orthopedic Surgery,Brigham and Women's Hospital Harvard Medical School Boston Massachusetts USA; ^2^ Operative Research Unit of Orthopaedic and Trauma Surgery Fondazione Policlinico Universitario Campus Bio‐Medico Rome Italy; ^3^ University Hospital Vienna Medical University of Vienna Vienna Austria; ^4^ Department of Orthopaedic and Trauma Surgery, University Medical Centre Mannheim, Medical Faculty Mannheim University of Heidelberg Mannheim Germany

**Keywords:** anterior cruciate ligament reconstruction, cartilage degeneration, patellofemoral osteoarthritis, post‐traumatic osteoarthritis

## Abstract

**Purpose:**

To investigate the development of patellofemoral osteoarthritis (PFOA) in the affected knee of anterior cruciate reconstruction (ACL‐R) patients compared to their contralateral knee. The impact of graft choice on PFOA progression was also examined.

**Methods:**

A systematic literature search was performed up to 1 June 2025. Studies at level of evidence II and III reporting imaging outcomes of the patellofemoral (PF) joint following ACL‐R and contralateral knees were included. Studies involving isolated primary ACL‐R procedures, regardless of meniscus status, were considered. The meta‐analysis was performed to assess if ACL‐R was associated with a higher likelihood of PFOA compared to contralateral knees. Furthermore, a sub‐analysis was conducted to evaluate whether the patellar tendon autograft was associated with a higher chance of PFOA than hamstring autografts. The random effects model was used to calculate the pooled odds ratio of PFOA in patients following ACL‐R compared to the control group. Meta‐regression analysis was performed to determine whether sample size, follow‐up duration and population age significantly influenced the odds ratio.

**Results:**

Eleven studies met the inclusion criteria and were included in this review. A total of 1206 participants were included, with an average male/female ratio of 57/49 and a pooled weighted mean age of 27 years. The follow‐up duration varied from 1 to 17.8 years, with an average of 5 years. Radiographic definitions of PFOA were determined using the Kellgren–Lawrence and the Osteoarthritis Research Society International (OARSI) classifications, while the magnetic resonance imaging (MRI) PFOA definition was derived from the MRI Osteoarthritis Knee Score (MOAKS) grading. Patients undergoing ACL‐R demonstrated a significantly higher likelihood of PFOA compared to their contralateral knees (*p* = 0.01). The use of patellar tendon or hamstring autografts did not show significant differences.

**Conclusions:**

Patients undergoing ACL‐R are more likely to develop PFOA than their contralateral knees. No significant difference in risk of PFOA development was found between hamstrings tendon and patellar tendon autografts.

**Level of Evidence:**

Level II and III, systematic review and meta‐analysis studies.

**Abbreviations**
ACL: anterior cruciate ligament
ACL‐R: anterior cruciate ligament reconstruction
BMI: body mass index
BPTB: bone patellar tendon bone
CI: confidence interval
HRQoL: health‐related quality of life
HT: Hamstring tendon
IKDC: International Knee Documentation Committee
I²: heterogeneity statistic
JSN: joint space narrowing
KL: Kellgren and Lawrence
KOOS: Knee Injury and Osteoarthritis Outcome Score
LET: lateral extra‐articular tenodesis
MCMS: Modified Coleman Methodology Score
MOAKS: MRI Osteoarthritis Knee Score
MRI: magnetic resonance imaging
OA: osteoarthritis
OARSI: Osteoarthritis Research Society International
OR: odds ratio
PF: patellofemoral
PFJ: patellofemoral joint
PFOA: patellofemoral osteoarthritis
PRISMA: Preferred Reporting Items for Systematic Reviews and Meta‐Analyses
PROMs: patient‐reported outcome measures
PROSPERO: prospective register of systematic reviews
PTOA: post‐traumatic osteoarthritis
RCT: randomised controlled trial
ROBINS‐I: Risk Of Bias In Non‐Randomised Studies – of Interventions
RoB 2: Risk of Bias 2 (Cochrane Tool for Randomised Trials)
SF‐36: 36‐Item Short Form Survey
TF: tibiofemoral
TFOA: tibiofemoral osteoarthritis

## INTRODUCTION

Anterior cruciate ligament reconstruction (ACL‐R) is commonly performed in young patients, particularly among athletes [35]. They undergo surgery to address instability, prevent concomitant injuries, and return to pre‐injury activity levels [35, 36]. Although ACL‐R is currently the therapeutic standard, studies have demonstrated that patients remain at risk of developing osteoarthritis (OA) [4, 6, 13]. The reported risk for developing OA 10–15 years after the injury has ranged widely from 16% to 90% [4, 23, 26]. Since ACL‐R patients are typically younger, OA occurs significantly earlier than in the general population [7, 37]. OA leads to a higher chance of joint replacement surgery than in comparable age cohorts [4, 7, 10, 21]. The emerging evidence suggests that, although less well‐recognised, patellofemoral OA (PFOA) deserves greater attention following anterior cruciate ligament (ACL) injury [10, 19]. PFOA contributes to knee symptoms after ACL injury, including anterior knee pain, swelling, and functional limitations like stair climbing and squatting. This early OA onset presents unique challenges for younger adults compared to older populations. The relationship between ACL‐R and PFOA remains unclear, but gaining more insight into it is crucial for improving functional outcomes. The present systematic review aims to evaluate the prevalence of PFOA in patients after ACL‐R compared to contralateral knees. It was also examined whether the choice of graft impacts PFOA progression. It is hypothesised that patients undergoing ACL‐R are more likely to develop PFOA than their contralateral knees. Furthermore, individuals receiving bone patellar tendon bone (BPTB) autografts had an increased likelihood of PFOA compared to individuals who received hamstring (HT) autografts.

## METHODS

A systematic literature search was conducted across the following databases: PubMed/MEDLINE, Embase, Cochrane Library and Scopus up to 1 June 2025. The MEDLINE search strategy was designed in consultation with a health sciences librarian and adapted for other databases to identify all studies that examined PFOA after ACL‐R.

The reproducible search string for PubMed/MEDLINE is as follows:

((‘patellofemoral osteoarthritis’ OR ‘patellofemoral arthritis’ OR ‘cartilage degeneration’ OR ‘cartilage deterioration’ OR ‘chondral lesion’ OR ‘cartilage loss’ OR ‘cartilage damage’ OR ‘osteoarthritis’) AND (‘anterior cruciate ligament reconstruction’ OR ‘ACL reconstruction’ OR ‘ACL surgery’ OR ‘anterior cruciate ligament injury’ OR ‘ACL tear’) AND (‘knee’ OR ‘knee joint’ OR ‘knee pain’ OR ‘knee instability’ OR ‘meniscus’ OR ‘biomechanics’ OR ‘gait analysis’ OR ‘rehabilitation’ OR ‘long‐term outcomes’ OR ‘MRI’ OR ‘radiographic’ OR ‘arthroscopy’ OR ‘arthroscopic surgery’ OR ‘risk factors’ OR ‘graft’ OR ‘hamstring tendon’ OR ‘bone‐patellar tendon‐bone (BPTB)’ OR ‘quadriceps’ OR ‘longitudinal study’ OR ‘clinical outcomes’ OR ‘surgical techniques’ OR ‘post‐operative care’ OR ‘functional outcomes’)).

The details are shown in Supporting Information: Table 1. Similar search strategies were used in Embase, Cochrane Library and Scopus.

The reporting in this systematic review was conducted according to the PRISMA guidelines [27].

### Eligibility

Only studies published in English that involved patients who had undergone primary ACL‐R with one or more years of follow‐up were included. To ensure robust and reliable outcomes, studies classified as level of evidence II and III, such as randomised controlled trials (RCTs), cohort studies, case‐control studies and cross‐sectional studies, were considered. Articles that reported only arthroscopic procedures and used either autograft or allograft for ACL‐R, with a minimum of 20 patients per group, were selected.

To maintain a clear focus for this review and minimise biases, studies involving procedures such as ACL repair, lateral extra‐articular tenodesis (LET), meniscal transplantations or cartilage repair interventions, as well as studies that included only patients receiving non‐operative treatment for ACL injury, were excluded. Lower‐quality studies (levels IV and V, including case series, case reports, expert opinions, cadaveric studies, in vitro, or animal studies) were not considered. Finally, studies that met the aforementioned criteria but did not provide clear radiographic or magnetic resonance imaging (MRI) data on PFOA were excluded. To maximise relevance to current‐day surgical and rehabilitation procedures, only studies published after 1999 were included.

### Study selection

Records were imported into a screening and data extraction platform (Cadima, www.cadima.info). Two investigators (D.F. and A.B.) reviewed the studies for the inclusion and exclusion criteria reported in Table 1. The full texts were reviewed if the titles and abstracts provided vague or insufficient information. The references of included studies were reviewed for additional sources. In case of any disagreement on the criteria, a third investigator (CBGL) was responsible for resolving the inconsistencies. All decisions, as well as inclusion and exclusion reasons, were recorded.

**Table 1:** Inclusion and exclusion criteria.

Inclusion criteria	Exclusion criteria
Patients of any age undergoing arthroscopic primary ACL reconstruction with autografts or allografts and 1 or more years of follow‐up	Patients undergoing ACL repair, lateral extra‐articular procedures
Randomised controlled trials, cohort studies and case‐control studies	Patients undergoing any concomitant procedures except meniscectomy or meniscal repairs
Including a control group comprised of contralateral knees	Case reports, reviews, expert opinions, cadaveric, in vitro and animal studies
Number of patients ≥20	Studies that do not report imaging outcomes regarding cartilage status or radiographic sign of PFOA
Studies published in English language	

Abbreviations: ACL, anterior cruciate ligament; PFOA, patellofemoral osteoarthritis.

### Data extraction

The data from valid studies were independently recorded in an Excel spreadsheet and validated through double‐checking. For each study included in this review, two reviewers (D.F. and A.B.) have extracted a range of data guided by Prognostic Factor Review Recommendations, including several study details such as title, authors, first author, journal, publication date, DOI, study design and level of evidence [32]. Moreover, there were extracted the following key data for analysis: participant characteristics, including age at the time of injury, sex ratio (male‐to‐female), preoperative body mass index (BMI), sample sizes at baseline and final follow‐up, the types of grafts used in the intervention; last follow‐up duration, definition of patellofemoral osteoarthritis (PFOA), encompassing the imaging outcome measures employed (e.g., radiographs, MRI) and specific thresholds or criteria for diagnosing PFOA; PFOA results, including the total number of affected knees in the experimental (injured) group, and those in the control (contralateral) group; clinical outcomes alongside any concurrent or subsequent injuries evaluated.

### Risk of bias and study quality assessment

The authors (D.F. and A.B.) independently assessed the risk of bias and the quality of the studies. Disagreements were resolved through consensus. The risk of bias was assessed for RCTs by Version 2 of the Cochrane risk‐of‐bias tool for randomised trials (RoB 2) and for non‐randomised cohort and case‐control studies by Risk Of Bias In Non‐Randomised Studies of Interventions (ROBINS‐I). The potential for selection, attrition, and measurement bias for PFOA outcomes and bias due to confounding and statistical analysis were categorised as low, with some concerns or moderate, and high or critical, based on signalling questions.

The quality of the studies was evaluated with the modified version of the Coleman Methodology Score (MCMS) [5].

### Data synthesis

The PFOA threshold value presented in the articles, in particular, after comparing the grading of different classification systems, was considered: Kellgren and Lawrence (KL) Grade ≥2, International Knee Documentation Committee (IKDC) Grade ≥B, Osteoarthritis Research Society International OARSI Grade ≥2 (or a sum of osteophyte grades of ≥2, or Grade 1 joint space narrowing (JSN) in combination with a Grade 1 osteophyte), MRI Osteoarthritis Knee Score (MOAKS) Grade ≥2. Two studies used an unconventional grade system for PFOA definition divided into three grades, and the X‐ray threshold for OA grade 1 or more was also considered about severity [2, 18].

Moreover, this review also considered studies that calculated the thickness in micrometers of the patella and trochlear cartilage using MRI to evaluate degenerative changes in cartilage, but not as raw data for meta‐analysis due to the limitations of usefulness. Finally, these different grading systems share similar severity of degenerative changes to define PFOA.

A meta‐analysis based on a random effects model was performed using R 4.4.1 (R Foundation for Statistical Computing, Vienna, Austria) to calculate the pooled odds ratio (OR) regarding the likelihood of PFOA in patients following ACL‐R compared to the control group. Sub‐analysis was conducted to assess the impact of graft choice on PFOA progression, and a meta‐regression was performed to determine whether sample size, follow‐up duration and population age significantly influenced the OR in this study. Heterogeneity tests were conducted and interpreted as follows: *I* ^2^ < 40%, low heterogeneity; 30% > *I* ^2^ < 60%, moderate heterogeneity; 50% > *I* ^2^ < 90% substantial, and *I* ^2^ > 75% to 100%, considerable heterogeneity [14]. In cases where PFOA was assessed at various time points in the same cohort, estimates from the last available follow‐up were included. Funnel plots were used to assess potential publication biases and study heterogeneity visually.

## RESULTS

### Study selection and characteristics

A total of 2829 studies were identified through the initial database search. After the removal of 32 duplicates, 2797 studies were screened. Of these, 2756 studies were excluded based on the title and abstract screening, and 73 full‐text articles were then identified and carefully read. After the full‐text assessment, 64 articles were excluded for various reasons: 29 studies lacked a control group, 6 studies involved only ACL injury and nonoperative treatment as a control group, 2 studies involved LET, 1 study focused on ACL repair, 13 studies had a sample size below 20 patients, 2 studies involved an open ACL‐R surgery and 11 studies did not specifically assessed PFOA.

**Figure 1:**
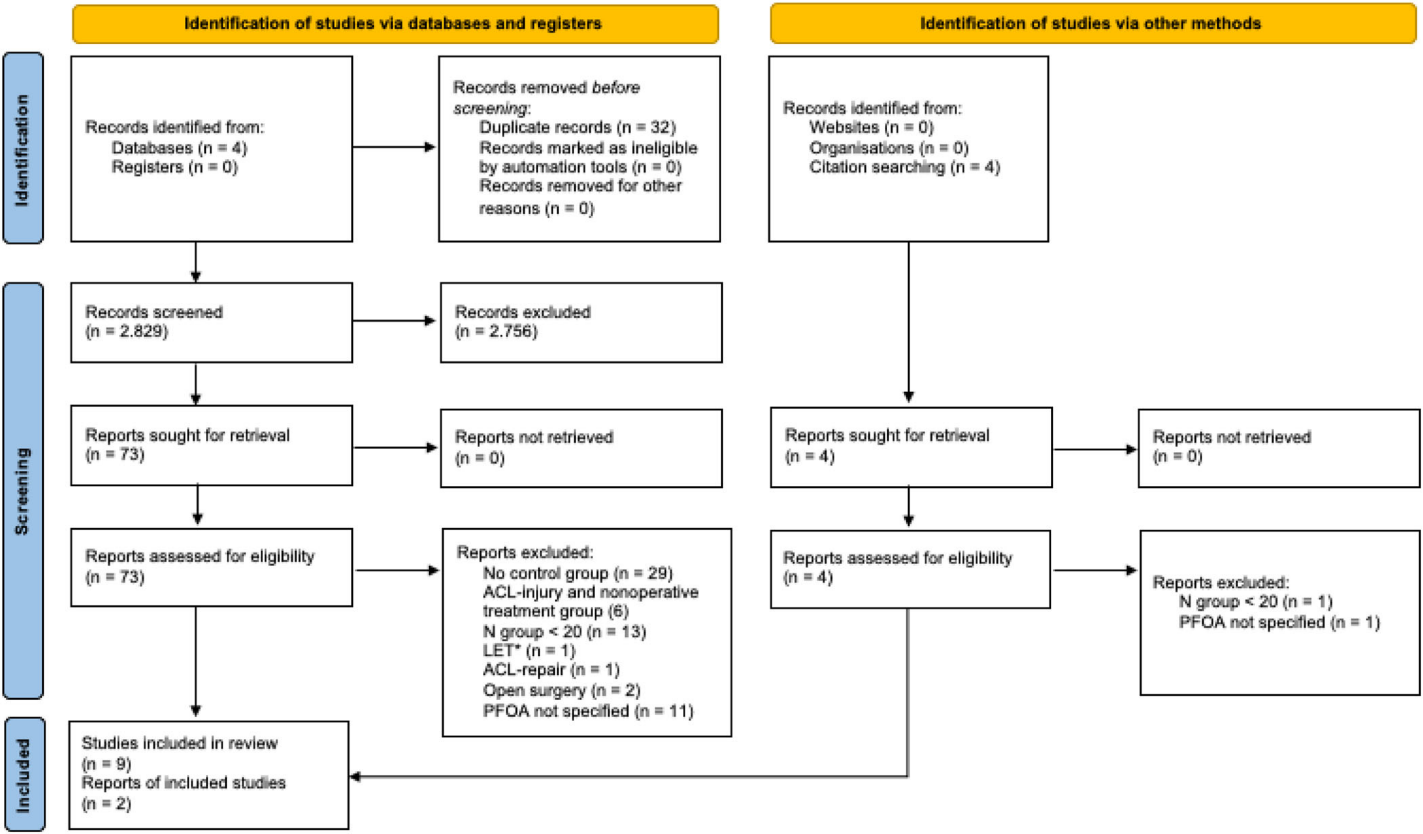
PRISMA (Preferred Reporting Items for Systematic Reviews and Meta‐analysis) flowchart of the study‐inclusion process. ACL, anterior cruciate ligament; LET, lateral extraarticular tenodesis; PFOA, patellofemoral osteoarthritis.

Ultimately, 11 studies met the inclusion criteria and were included in the systematic review (Figure 1).

These 11 studies considered a total of 1206 participants (1094 patients with ACL‐R, 1093 with contralateral knee) from 11 unique cohorts. The included studies consisted of three RCTs [1, 2, 16], seven cohort studies [8, 18, 22, 28, 31, 33, 39] and one case‐control study [20], published between 2004 and 2023. Regarding the graft choice, the autologous BPTB was used in three articles [16, 28, 31], the autologous HT were used in one article [8], BPTB and HT in different combinations were used in six articles [1, 2, 18, 20, 33, 39] and allograft was used in two studies [20, 39]. An Achilles tendon autograft was utilised in one study [20]. In one article, the graft choice used was not specified: it mentioned the use of BPTB graft in 65% of cases, while in the remaining 35% of cases, the graft type was not clarified [22]. The average male/female ratio of the included studies was 57/49 and the pooled weighted mean age was 27 years. The follow‐up period ranged from 1 [8] to 17.8 years [33], with a pooled mean follow‐up period of 5 years. Three studies [8, 18, 31] used both contralateral knees and healthy individuals as control groups but only the contralateral knees were considered in the analysis. In contrast, another study included both contralateral knees and non‐operatively treated ACL injury patients as controls, but only the contralateral knee group was included in this article [22].

A total of seven studies were considered in the meta‐analysis. Four studies included in the systematic review were not considered for the meta‐analysis. Two studies were excluded due to a lack of clear and quantifiable radiographic/MRI PFOA outcomes necessary for statistical inclusion [31, 39]. However, they met the established inclusion criteria for this systematic review. Although the study's contribution is limited regarding the extent of PFOA following ACL‐R, they still provide evidence of patellofemoral (PF) cartilage degeneration [31, 39]. Moreover, one study used contralateral knees as a control group but did not specify how many were affected by PFOA [16]. Finally, one study considered the contralateral knees only for clinical assessment rather than PFOA outcomes [18]. The demographic characteristics of the included studies are summarised in Table 2a.

**Table 2a:** Demographic characteristics of the studies included in the systematic review.

Study design	Level of evidence	Sex (M/F)	Age (mean ± SD)	BMI (kg/m^2^) ± SD	*N* (study)	Type of control group	*N* (control)	*N* (intervention)	Graft Type	Follow‐up time (years)
Cohort study	III	28/29	20.3 ± 5.12	23.9 ± 3.64	57	Contralateral knee	57	57	BPTB (autograft)	2.9
Case–control study	III	153/96	25.6 ± 11.4	25.4	249	Contralateral knee	249	249	Autograft: hamstring vs (BPTB/Achilles); Allograft	7.8
Chort study	III	16/14	22.1 ± 1.4	NA	30	Contralateral knee	30	30	15 BPTB (50%), 9 Hamstring (30%), 6 Allograft (20%)	2.5
Cohort study	III	50/49	29.1 ± 9.66	NA	99	Healthy control group; contralateral knee;	Healthy patients: 46; contralateral knee: 53	53	BPTB (autograft)	3.8
Randomised controlled trial	II	95/52	21.6 ± 11.4	NA	143	Contralateral knee	143	142	Hamstring tendon (83), BPTB (59)	16
Randomised controlled trial	II	55/55	26.4 ± 6.41	NA	135	Contralateral knee	135	134	69 BPTB 65 hamstring	14
Cohort study	III	84/47	28 ± 8	26 ± 5	131	Healthy control group; contralateral knee;	Contralateral knees: 111; Healthy controls: 20	111	Hamstring tendon (autograft)	1
Cohort study	III	0/103	46 ± 6	23	67	ACL‐injury + rehab; contralateral knee;	AACL‐injury + rehab (26); contralateral knee (41)	41	BPTB 65%, no mentioned 35%	12
Cohort study	III	95/73	27.4 ± 9.1	NA	168	Contralateral knee	167 contralateral knee; 1 exclused pregnant	168	86% BPTB autograft (*n* = 144) 14% quadrupled hamstring tendon autograft (*n* = 24)	17.8
Randomised controlled trial	II	41/26	27.9 ± 7.8	NA	53	Contralateral knee	53	53	BPTP (autograft): 25 open ACL‐R; 28 arthroscopic ACL‐R	12
Cohort study	III	44/18	27	NA	74	Healthy control group; contralateral knee;	18 healthy patients; 56 contralateral knee (clinical analysis)	56	Autograft: 27 hamstring; 28 BPTB	6

Abbreviations: ACL injury + rehab, anterior cruciate ligament injury group with non‐operative treatment; BMI, body mass index; BPTB, bone patellar tendon bone; HT, hamstring tendon; *N* (study), number of patients included in the study; *N* (control), number of contralateral knees included in the study; *N* (intervention), number of patients who undergone anterior cruciate ligament reconstruction.

### Image outcomes

Radiographic definitions of PFOA were based on a variety of grading systems, including the KL (*n* = 5) [1, 16, 20, 22, 33] and OARSI Atlas (*n* = 1) [8]. The MRI PFOA definition was based on MOAKS (*n* = 2) grading [8, 28]. Two articles did not specify any image definition score of PFOA but provided evidence of MRI cartilage degeneration after ACL‐R [31, 39]. Two studies implemented a different grading system for PFOA definition, categorising it into four grades (none, mild, moderate and severe), and the threshold for OA grade 1 or higher in terms of severity was considered [2, 18].

### Prevalence of patellofemoral osteoarthritis

**Figure 2:**
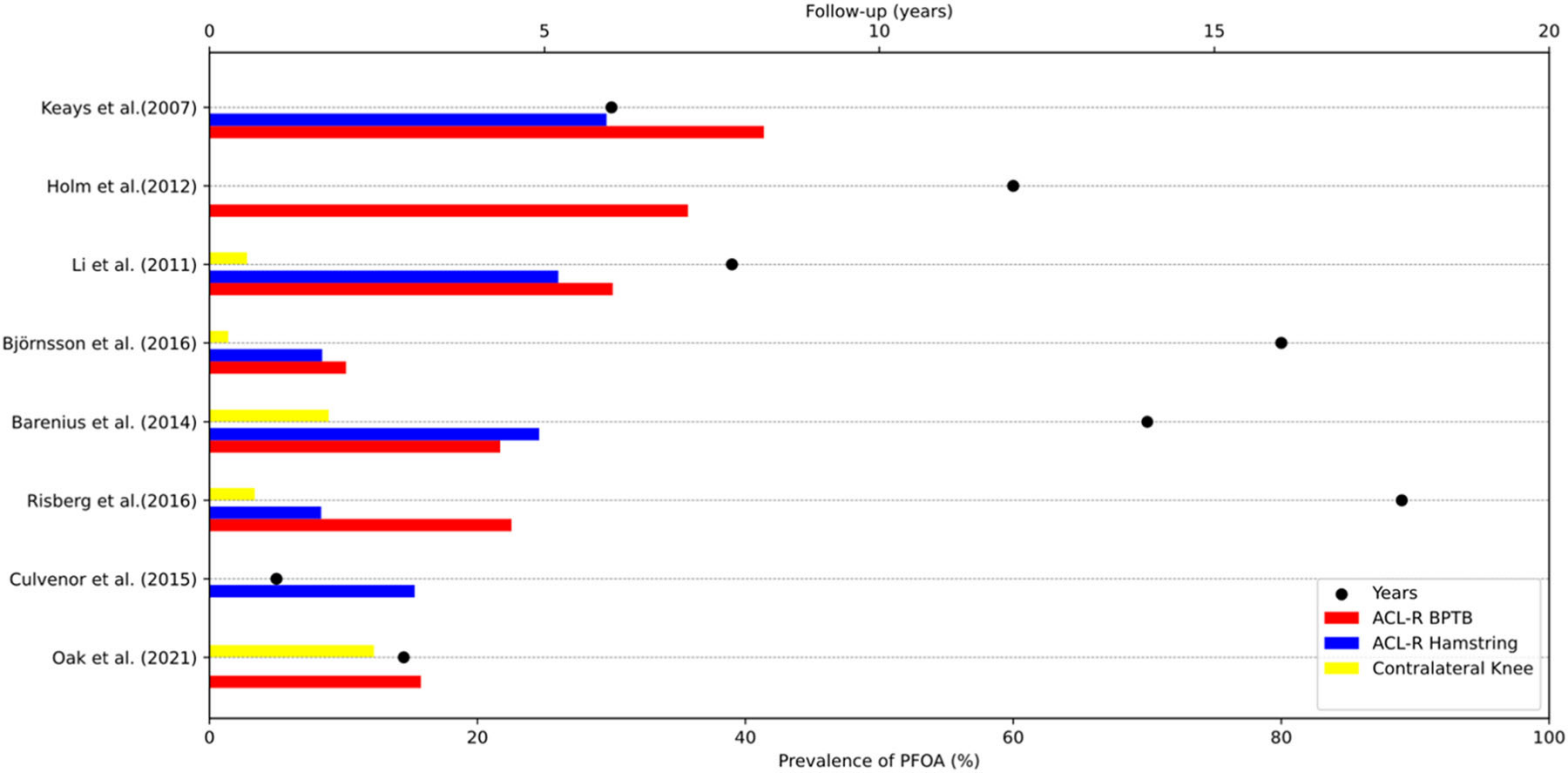
Patellofemoral osteoarthritis prevalence plot of anterior cruciate ligament‐reconstructed patients based on graft used and contralateral knees. ACL‐R, anterior cruciate ligament reconstruction; BPTB, bone patellar tendon bone; PFOA, patellofemoral osteoarthritis.

The pooled prevalence of PFOA in those who underwent ACL‐R was 21.9%, with a range from 9.2% to 35.7%. The overall prevalence in the contralateral knee groups was 4.1%, ranging from 0% to 12.3%. In the ACL‐R population with BPTB autograft, the overall prevalence of PFOA from seven studies was 24.2%, ranging from 10.2% to 41.4%. On the other hand, when ACL‐R was performed with HT autograft, the overall prevalence of PFOA from six studies was 18.5% (8.3%–29.6%; Figure 2). The ACL‐R group with BPTB autograft consisted of 453 patients with an average follow‐up of 10.4 years. In contrast, the ACL‐R group with HT autograft comprised 319 patients with an average follow‐up of 9.6 years.

Clinical and patient reported outcomes (PROMs) were presented in 10 articles, in particular: six studies [1, 22, 28, 31, 33, 39] considered Knee Injury and Osteoarthritis Outcome Score (KOOS). The Lyshom score was used in two studies [2, 16], one study considered the Cincinnati knee score [16] and Tegner score was assessed in two studies [1, 2]. The International Knee Documentation Committee score (IKDC) was evaluated in two articles [2, 31] and HRQoL in one article [1]. Three studies showed no PROMs [8, 18, 20]. Regarding the presence of concomitant injuries, meniscus tears were described in four articles [1, 2, 28, 33] and meniscal procedures, such as meniscectomy or meniscus repairs, were reported in seven articles [8, 16, 18, 20, 22, 31, 39]. The image‐based and clinical characteristics of the included studies are summarised in Table 2b.

**Table 2b:** Image‐based and clinical outcomes of the included studies.

Study	Radiographic outcome measure	Threshold OA (X‐ray)	Results of radiographic outcome	MRI outcome measure	Threshold OA (MRI)	Results of MRI outcome	PROM	Additional injury number or rate
Oak et al. [28]	NA	NA	NA	MOAKS	Presence of a full‐thickness cartilage loss in the PFJ	Number of PFOA patient after ALC‐R: 9 Number of total patients undergone ACL‐R: 57 Number of contralateral knee with PFOA: 7 Number of total contralateral knee: 57	KOOS	31 medial meniscus tears; 16 lateral meniscus tears
Li et al. [20]	KL	KL ≥ 2	Ipsilateral knee: PFOA: 71 out of 249 patients (11.2%) Contralateral knee: PFOA: 7 out of 249 patients (2.8%)	NA	NA	NA	NA	OA patients: – Concurrent lateral meniscectomy: 19; concurrent medial meniscectomy: 24; prior lateral meniscectomy: 7; prior medial meniscectomy: 9 Non‐OA patients: – Concurrent lateral meniscectomy: 22; concurrent medial meniscectomy: 13; prior lateral meniscectomy: 0; prior medial meniscectomy: 3
Williams et al. [39]	NA	NA	NA	T2 relaxation times for patellar and trochlear cartilage	Prolonged patello‐femoral T2 relaxation time in injured knee compared to contralateral knee	Longer T2 relaxation time in the operated knee compared to contralateral knee (trochlea). No significant difference in patellar cartilage	KOOS	Meniscectomy: 14
Pius et al. [31]	NA	NA	NA	Cartilage thickness	Not present Difference between injured knee (ACL‐R) and contralateral knee vs. healthy subjects	Patella Females: 30% thickening (mean 0.28 mm); 3% thinning (mean −0.37 mm). Males: 4.6% thickening (mean 0.28 mm); a small area showed thinning (mean −0.63 mm). Trochlea Females: 25.6% thickening (mean of 0.29 mm); 4.2% thinning (mean of −0.24 mm). Males: 2.3% thickening (mean of 0.09 mm); 2.3% thinning (mean of −0.53 mm).	KOOS, IKDC score	17 medial menisci treated (13 repairs, 4 partial meniscectomies) 27 lateral menisci treated (3 repairs, 24 partial meniscectomies)
Björnsson et al. [2]	NOT SPECIFIC classification for PFOA. OA changes in the PF joint were classified as none (0), minor [1], moderate [2], or severe [3].	≥1	PFOA: BPTB group Injured knee: Grade 0 (no arthritis): 51 patients; grade 1 (mild arthritis): 7 patients; grade 2 (moderate arthritis): 1 patient; grade 3 (severe arthritis): 0 patients Contralateral knee: Grade 0: 54 patients; Grade 1: 1 patient; Grade 2: 3 patients; Grade 3: 1 patients HT group Injured knee: Grade 0: 78 patients; grade 1: 3 patients; Grade 2: 1 patient; Grade 3: 1 patient Contralateral knee: Grade 0: 80 patients; Grade 1: 1 patient; Grade 2: 2 patients; Grade 3: 1 patient;	NA	NA	NA	Lysholm score, Tegner score, IKDC score	Meniscal injuries were observed in 69 participants (57%) on baseline MRI; No specific check with radiographic evaluation
Barenius et al. [1]	KL	KL ≥ 2	HT group (contralateral knee): 6 patients HT group (primary ACL‐reconstructed knee): 13 patients BPTB group (contralateral knee): 6 patients BPTB group (primary ACL‐reconstructed knee): 20 patients	NA	NA	NA	Tegner score, HRQoL, KOOS	Meniscus Injury – Medial meniscus: BPTB Group: 32 (46%); ST Group: 24 (37%) – Lateral meniscus: BPTB Group: 17 (25%); ST Group: 26 (40%) Meniscus Procedure – Medial meniscus resection: BPTB Group: 23 (33%); ST Group: 19 (29%) – Lateral meniscus resection: BPTB Group: 10 (15%); ST Group: 21 (32%) – Medial meniscus suture: BPTB Group: 10 (15%); ST Group: 4 (6%) – Lateral meniscus suture: BPTB Group: 3 (4%); ST Group: 4 (6%) Meniscus Procedures After ACL‐R BPTB Group: 6 (9%); ST Group: 5 (8%)
Culvenor et al. [8]	OARSI atlas	Any of the following criteria: JSN ≥ grade 2; Sum of osteophyte grades ≥ 2; Grade 1 JSN in combination with grade 1 osteophyte	• Radiographic PFOA in ACL‐R group: 2% (2/111)• Radiographic PFOA in control group: 0% (0/20)	MOAKS	Participants were classified as having PFOA on MRI if they had a definite osteophyte and partial‐ or full‐thickness cartilage loss in the PF compartment. We considered a definite osteophyte to be present when the sum of osteophyte grades in a single compartment was ≥2, consistent with radiographic criteria.	PF MRI‐OA: ACL‐R Group: 16% (17/111) Contralateral knees: 0% (0/111) Healthy controls: 0% (0/20) TF MRI‐OA: ACL‐R Group: 19% (21/111) Control Group: 0% (0/20) Bone Marrow Lesions (BMLs): Femoral trochlea: 19% of ACL‐R participants Cartilage Lesions: Femoral trochlea: 31% of ACL‐R participants Osteophytes: Femoral trochlea: 37% of ACL‐R participants	NA	Meniscus surgery: 23 medial meniscectomy; 23 lateral meniscectomy
Lohmander et al. [22]	KL	KL ≥ 2	PFOA: – 8 (20%) in ACL reconstructed knees – 1 (4%) in ACL‐injury + rehab; 0 contralateral knee	NA	NA	NA	KOOS	– Meniscus surgery: 40% (34/84)
Risberg et al. [33]	KL	KL ≥ 2	Involved (operated) knee: Number of patients with PFOA: 35 patients (21%); 11 patients (7%) have isolated PFOA. PFOA was more common in patients with combined injuries (meniscus and/or cartilage) compared to those with isolated ACL‐injury. Contralateral (non‐operated) knee: Number of patients with PFOA: 9 patients. The prevalence of PFOA in the contralateral knee is significantly lower compared to the involved knee.	NA	NA	NA	KOOS; Tegner score	63% of subjects had combined injuries; 37% had isolated ACL injury
Holm et al. [16]	KL	KL ≥ 2	Radiographic patellofemoral OA: 10 arthroscopic BPTB; Not mentioned contralateral knee	NA	NA	NA	Cincinnati knee score, Lysholm score, VAS	Meniscal surgery: ACL‐R group: 16 patients
Keays et al. [18]	NOT SPECIFIC classification for PFOA. OA changes in the PF joint were classified as none (0), mild(1), moderate (2), or severe (3).	≥1	PFOA in ACL reconstructed knees: 8 Knees with Hamstring; 12 Knees with BPTB	NA	NA	NA	NA	Meniscectomy: BPTB group, 23 patients. Hamstring group, 20 patients.

Abbreviations: ACLOS, Anterior Cruciate Ligament OsteoArthritis Score; ACL injury + rehab, anterior cruciate ligament injury group with non‐operative treatment; BPTB, bone patellar tendon bone; HRQoL, health‐related quality of life; HT, Hamstring tendon; IKDC score, the International Knee Documentation Committee score; JSN, joint space narrowing; KL, Kellgren–Lawrence classification; KOOS, the Knee Injury and Osteoarthritis Outcome Score; MOAKS, MRI Osteoarthritis Knee Score; MRI, magnetic resonance imaging; OA, osteoarthritis; OARSI atlas, Osteoarthritis Research Society International Classification; PF, patellofemoral; PFJ, patellofemoral joint; PFOA, patellofemoral osteoarthritis; SF‐36, 36‐Item Short Form Survey.

### Risk of bias and methodological quality

**Figure 3:**
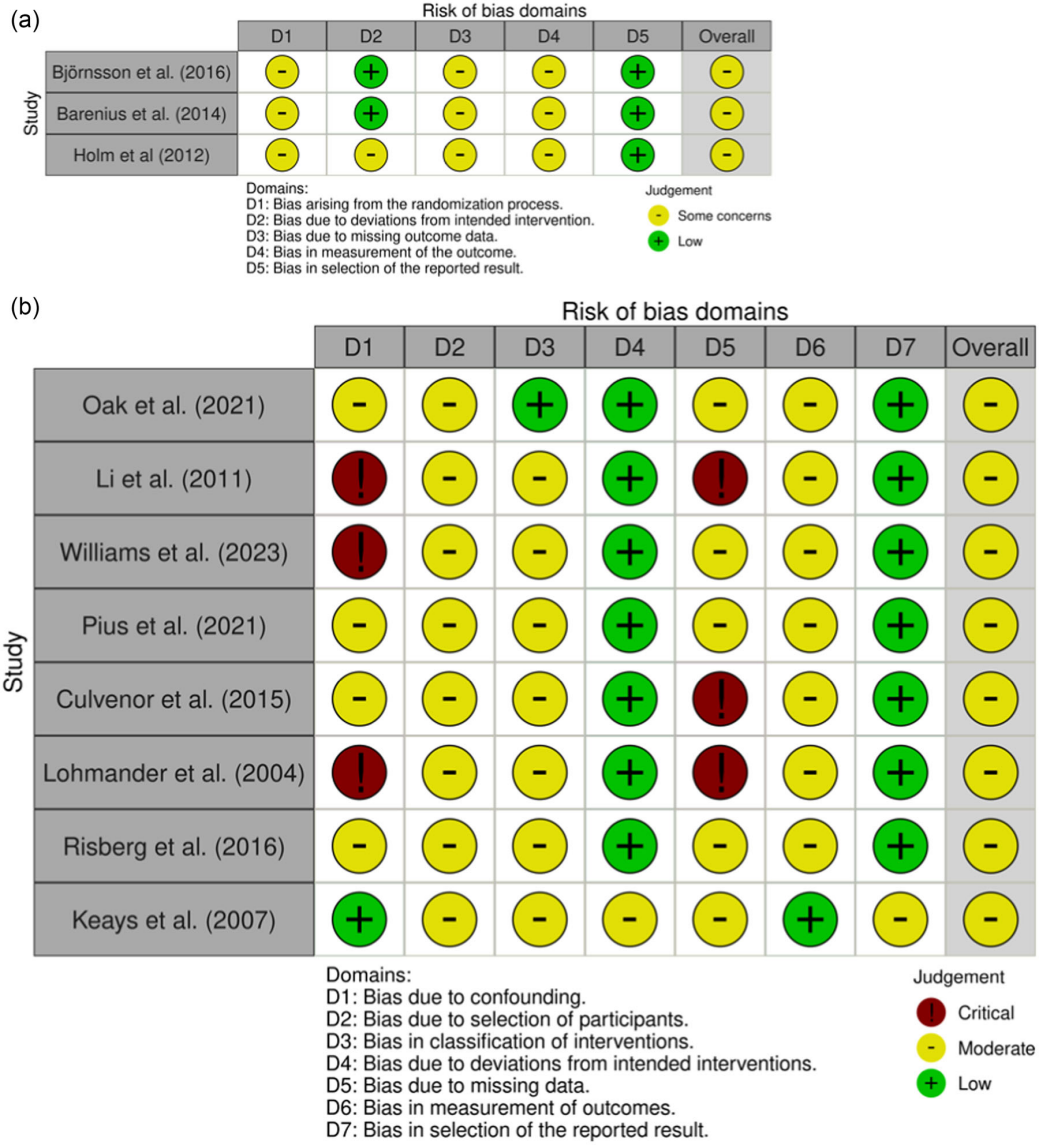
Risk of bias for included randomised controlled trials (a) and non‐randomised controlled trials (b).

The risk of bias for the included RCTs was assessed using the RoB 2 tool. All studies had a moderate risk of bias, largely due to issues related to randomisation, missing outcome data, and the lack of clear outcome measurement. The risk of bias for non‐randomised studies was assessed using the ROBINS‐I, demonstrating an overall moderate risk of bias. The results showed several biases in areas related to confounding and missing data. The risk of bias assessment is summarised in Figure 3.

Regarding methodological quality, the MCMS [5] was applied to all included studies, with scores ranging from 67 to 83.

Overall, the average MCMS score across all included studies was 76, indicating a moderate level of methodological quality. The most common limitations identified were the lack of clarity in the description of rehabilitation protocols and variability in the number of surgical procedures (Table 3).

**Table 3:** Modified Coleman Methodology Score (MCMS).

	Part A							Part B		Part A			
References	Study size	Follow‐up	Number of procedures	Study type	Diagnostic certainty	Description of procedure	Description of rehabilitation	Outcome criteria	Outcome assessment	Selection process		Part B	Tot. MCMS
Oak et al. [28]	7	5	10	5	5	10	5	10	7	15	47	32	79
Li et al. [20]	7	5	0	5	5	10	10	10	8	15	42	33	75
Williams et al. [39]	4	5	0	5	5	10	5	10	8	15	34	33	67
Pius et al. [31]	10	5	10	5	5	10	5	8	10	15	50	33	83
Björnsson et al. [2]	10	5	0	10	5	10	5	10	10	15	45	35	80
Barenius et al. [1]	10	5	0	10	5	10	5	8	10	15	45	33	78
Culvenor et al. [8]	10	2	10	5	5	5	5	8	10	15	42	33	75
Lohmander et al. [22]	10	5	0	5	5	5	5	8	11	15	35	34	69
Risberg et al. [33]	10	5	0	5	5	10	5	8	10	15	40	33	73
Holm et al. [16]	4	10	0	10	5	10	10	7	12	10	49	29	78
Keays et al. [18]	10	5	10	5	5	10	5	7	12	10	50	29	79

### Meta‐analyses

**Figure 4:**
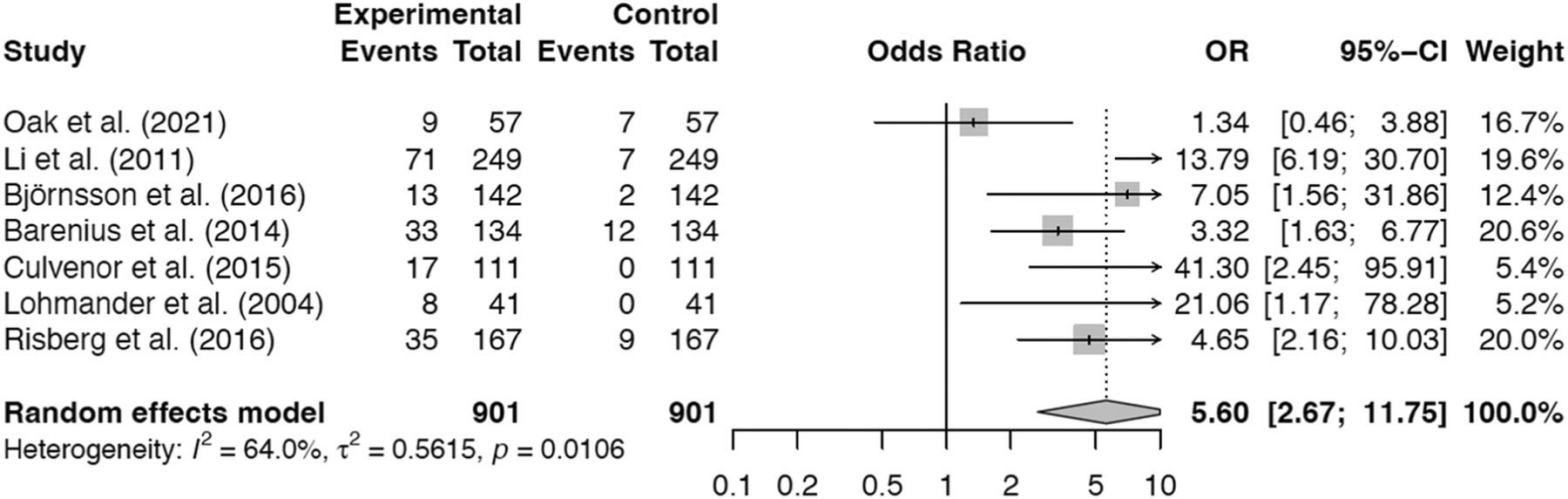
Forest plot for the prevalence of patellofemoral osteoarthritis following anterior cruciate ligament (ACL) reconstruction compared with contralateral knees. CI, confidence interval; *I* ^2^, heterogenety; OR, odds ratio; *p*, *p* value; τ^2,^ Tau square.

**Figure 5:**
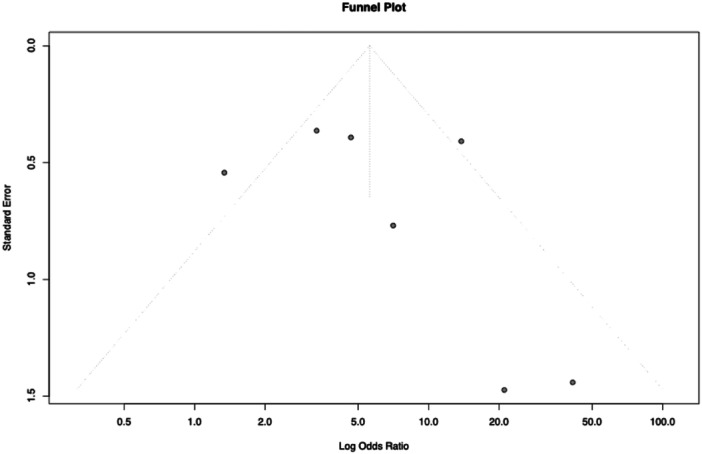
Funnel plot of odds ratios for the prevalence of patellofemoral osteoarthritis post‐anterior cruciate ligament reconstruction.

The odds ratio (OR) for the development of PFOA was significantly higher in patients who underwent ACL‐R compared to a control group of contralateral knees (pooled OR 5.60, 95% CI: 2.67–11.75, *p* = 0.01). There was moderate heterogeneity across the included studies (*I*² of 64.00%; Figures 4 and 5).

In the comparison between BPTB and HT autografts, no significant difference was found between the type of graft and the likelihood of PFOA progression (*p* = 0.77) (Supporting Information: Figures 1 and 2).

The meta‐regression revealed that sample size influences the analysis outcome (*p* = 0.004). The significance of the moderator test (*p* = 0.006) further validated the regression model. In contrast, follow‐up duration and mean age did not emerge as influential factors.

## DISCUSSION

The main findings of this study are that the prevalence of PFOA following ACL‐R varies widely across the included studies, ranging from 9.2% to 35.7%. ACL‐R was associated with a pooled OR of approximately fivefold for the development of PFOA compared to contralateral knees, reinforcing that ACL‐R does not protect against PFOA development.

The sub‐metanalysis comparing BPTB and HT autograft for ACL‐R did not reveal statistically significant differences, underscoring the equal chance of developing PFOA.

The findings of this review are consistent with previous articles, such as those by Chen et al. [3] and Øiestad et al. [29], which also identified an increased prevalence of PFOA following ACL‐R [17, 29].

Previous studies have also reported the highest association with degenerative changes in the PF joint following ACL‐R when BPTB grafts are used [11, 20]. In contrast, Lucidi et al. found no significant increase in PF joint degeneration with BPTB grafts compared to other graft types at 20‐year follow‐up [24]. This systematic review did not find statistical significance regarding the incidence of PFOA in patients who underwent ACL‐R with the BPTB autograft. However, the meta‐regression revealed that sample size influences the outcome of the analysis. These findings suggest the lack of statistical significance in the sub‐analysis may be attributed to insufficient sample size, highlighting a potential issue of low statistical power and warranting further investigation. This finding will be particularly important for patients with pre‐existing PF chondral wear at the time of injury, as it could influence graft choice for the ACL‐R.

This study could not determine whether BMI plays a role in increasing PFOA after ACL‐R due to the absence of a clear BMI assessment in the studies. However, previous articles have indicated potential risk factors in the biomechanical and inflammatory pathways that may contribute to increased cartilage wear and osteoarthritic changes, particularly in the PF component after ACL‐R [12]. These results highlight the necessity of further studies for considering BMI as a modifiable risk factor in the management and prognosis of ACL‐injury patients.

The prevalence of PFOA following ACL‐R was 18.4% ranging from 9.2% and 35.7% for prospective studies [1, 2, 16]. Moreover, the prevalence varied between 15.8% and 35.7% for the retrospective studies [9, 18, 20, 22, 28, 33]. The PFOA prevalence, as reported by studies comparing the influence of graft choice, ranged from 8.3 to 29.6% for HT autograft and from 10.2% to 41.4% for BPTB autograft.

Additionally, the contralateral knee group reported a prevalence of PFOA ranging from 0% to 12.3%. Compared with the 1% to 2% prevalence of overall knee OA reported in the general age‐matched population without ACL injuries [8, 34], this variation could suggest altered joint loading in the contralateral knee after ACL injury and reconstruction. The variation in the reported prevalence of PFOA for the included studies is probably owing to several factors: different study designs and weak methodological quality, different graft choices, surgical techniques, and associated procedures, as well as different radiologic classification systems used to evaluate knee PFOA [23].

The different study populations (subjects with isolated ACL injury vs combined injuries, in particular preoperative cartilage damage, follow‐up period, and subjects with different types and levels of sports activities) could probably also explain some of the variations and make it challenging to compare all the study results.

Finally, the data extracted in this systematic review and meta‐analysis revealed a tendency indicating that ACL‐R as a single factor will not prevent the development of knee PFOA and also increase the likelihood of degenerative changes in the PF joint. PFOA today is likely an under‐recognised outcome of ACL‐R, even though it appears to be at least as common as tibiofemoral OA [7]. These findings are crucial as they emphasise the need for careful ACL‐R surgical planning to mitigate the risk of PFOA, especially in patients who already have PF degeneration. Furthermore, early post‐operative interventions, such as tailored rehabilitation programs, may be necessary to reduce the degenerative processes on the PF joint [15, 30].

This meta‐analysis focused on analysing ACL‐R patients and contralateral knees. All previous reviews did not consider any control group [4, 17, 25, 29]. This approach allowed us to establish a clearer association between ACL‐R and the likelihood of developing PFOA, reducing biases related to the individual risk of PFOA inherent in each patient.

Several limitations must be acknowledged for this study. First, although the strict inclusion criteria enhanced the quality of the study, they also led to a smaller pool of eligible studies, which may limit these findings. Inevitable variables of the included studies, such as patient activity levels, rehabilitation protocols, return to sport after surgery, concomitant contralateral injury, and diagnostic criteria for PFOA, could significantly influence the results.

The data are combined with heterogeneity across different population ages and follow‐up times. Data from early time points when structural degenerative changes in the knee are less prevalent may underestimate the odds of PFOA.

On the other hand, the stringent inclusion criteria exclude studies without an adequate control group and eliminate potential major confounding factors, thereby increasing the overall quality of the included studies.

Further investigations into modifiable risk factors, such as BMI, graft selection and rehabilitation strategies, could provide insights into reducing the prevalence of PFOA in ACL‐R patients. Broader studies examining the long‐term effects of various ACL‐R surgical techniques on PF joint mechanics and biology may provide strategies to enhance surgical outcomes and decrease the occurrence of post‐operative PFOA.

As previously mentioned, prior studies have associated the BPTB graft with an increased risk of degenerative changes in the PF joint. Although this trend did not reach statistical significance in this study, possibly due to a limitation in power analysis, the direction suggests a possible future association. While speculative, this may be related to the BPTB autograft affecting the extensor mechanism [38]. If this hypothesis is correct, the quadriceps tendon autograft should also be scrutinised concerning PFOA development. In this review, only one of the included studies used quadriceps tendon autografts but the prevalence of PFOA was not assessed. In any case, a single study may not be representative, and investigations are also needed to assess PFOA more thoroughly in cohorts of patients undergoing ACL‐R with quadriceps tendon autografts.

The study by Williams et al. showed the longer T2 relaxation time on MRI that was identified as an important factor in analysing the cartilage status of the PF joint, and the time sequence was significantly longer in the operated knee compared to the contralateral knee [39]. However, the exact number of patients showing this deterioration was not directly specified, and no specific cut‐off was provided to identify PFOA. Additionally, Pius et al. [31] analysed the changes in articular cartilage thickness following ACL‐R, focusing on sex‐based differences. The study offered valuable insights into cartilage's thickening and thinning patterns, comparing the results with those of contralateral knees. However, it did not provide a specific threshold for defining PFOA's presence, making it challenging to include in the quantitative synthesis [31].

## CONCLUSION

This study demonstrates an association between ACL‐R and PFOA, showing that patients undergoing ACL‐R are likelier to develop PFOA than contralateral knees. The sub‐analysis comparing BPTB and HT autograft for PFOA progression following ACL‐R did not reveal statistically significant differences. Future strategies are essential to mitigate the individual risk of PFOA following ACL‐R.

## AUTHOR CONTRIBUTIONS

*Conceptualisation*: Domenico Franco. *Methodology*: Domenico Franco and Alexander Bumberger. *Formal analysis and investigation*: Domenico Franco and Alexander Bumberger. *Writing draft preparation*: Domenico Franco. *Review*: Alexander Bumberger, Chilan B.G. Leite, Sebastian Schmidt, Rocco Papalia, Vincenzo Denaro, Cale Jacobs, and Christian Lattermann. *Editing*: Domenico Franco. *Supervision*: Christian Lattermann.

## CONFLICT OF INTEREST STATEMENT

The authors declare no conflicts of interest.

## ETHICS STATEMENT

The study was registered on PROSPERO with ID: CRD42024571939 on 22 July 2024.

## Supporting information

Supporting information.
