# Supplementary material for: Elevated risk of patellofemoral osteoarthritis following ACL reconstruction compared to contralateral knees: A systematic review and meta‐analysis
Source: J Exp Orthop. 2025 Nov 18;12(4):e70467. doi: 10.1002/jeo2.70467 (PMC12626278; doi:10.1002/jeo2.70467)
Supplement: Supplementary file 1 — Supporting information. [file JEO2-12-e70467-s001.docx]

**SUPPLEMENTAL MATERIAL**

**Supplementary Table 1. Search terms.**

**
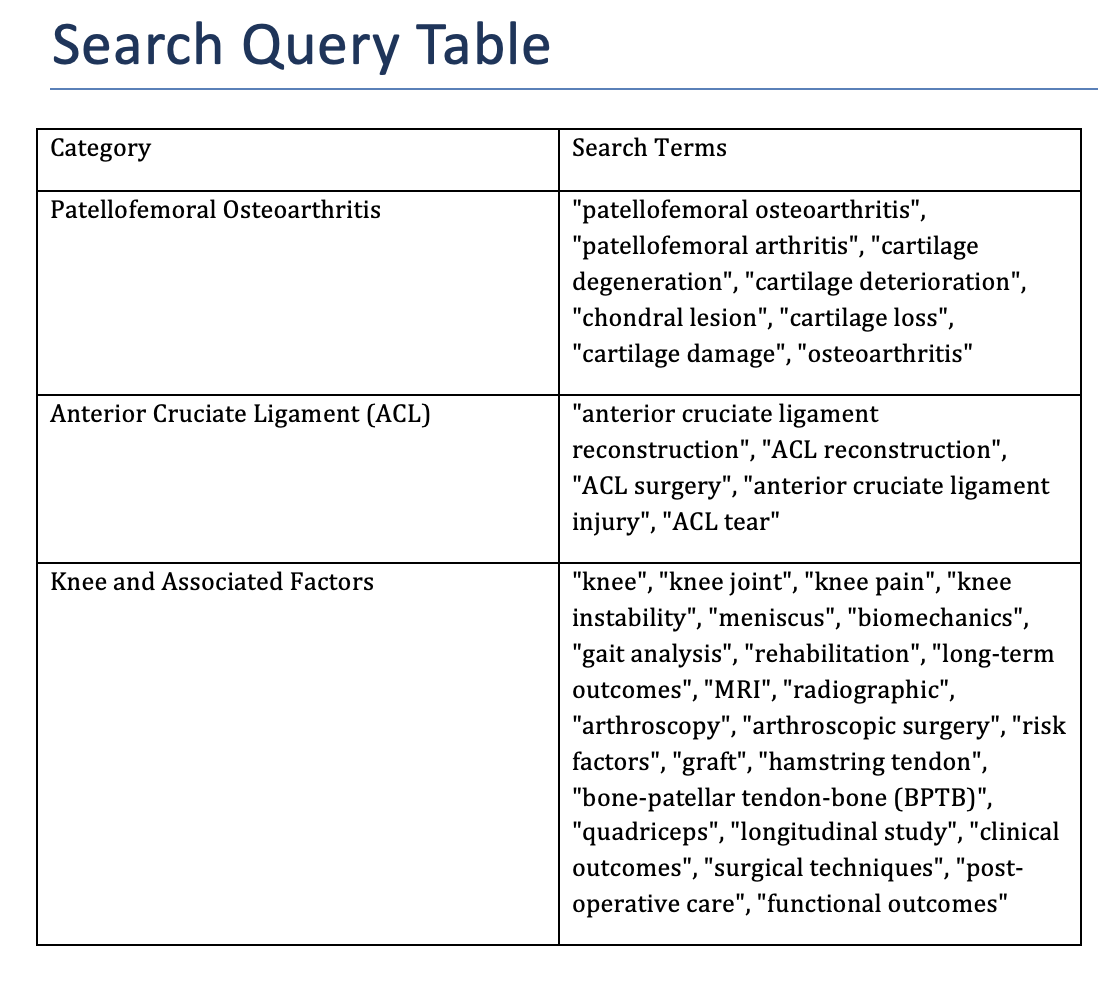
**

(Patellofemoral osteoarthritis OR patellofemoral arthritis OR cartilage degeneration OR chondral lesion OR cartilage loss OR cartilage damage OR osteoarthritis) AND (anterior cruciate ligament reconstruction OR ACL reconstruction OR ACL surgery OR anterior cruciate ligament injury OR ACL tear) AND (knee OR knee joint OR knee pain OR knee instability OR meniscus OR biomechanics OR gait analysis OR rehabilitation OR long-term outcomes OR MRI OR radiographic OR arthroscopy OR arthroscopic surgery OR risk factors OR graft OR hamstring tendon OR bone-patellar tendon-bone OR quadriceps OR longitudinal study OR clinical outcomes OR surgical techniques OR post-operative care OR functional outcomes)


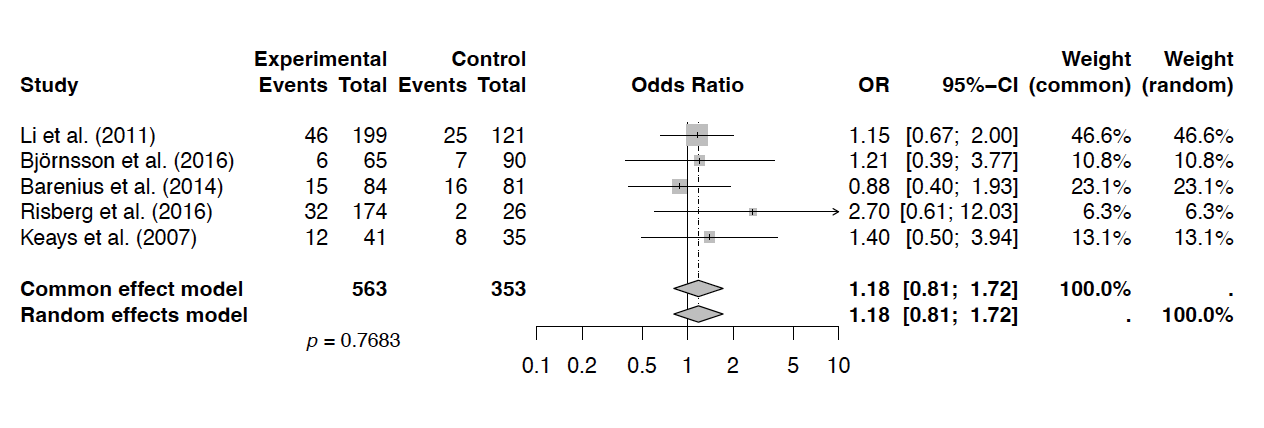


Supplementary Fig.1 **Forest Plot for the Prevalence of Patellofemoral Osteoarthritis Following Anterior Cruciate Ligament (ACL) Reconstruction between bone-patellar tendon-bone and Hamstring groups.**

OR= Odds Ratio; CI= Confidence Interval; p = P value;

Supplementary Fig.2 **Funnel Plot of Odds Ratios for the Prevalence of Patellofemoral Osteoarthritis Post-ACL Reconstruction between bone-patellar tendon-bone and Hamstring groups.**
